# The Correlation Between ABPM Parameters and Left Ventricular Hypertrophy in Pediatric Essential Hypertension

**DOI:** 10.3389/fped.2022.896054

**Published:** 2022-06-02

**Authors:** Haojie Wu, Lin Shi, Yao Lin, Tong Zheng

**Affiliations:** ^1^Capital Institute of Pediatrics, Beijing, China; ^2^Department of Cardiology, Children’s Hospital, Capital Institute of Pediatrics, Beijing, China

**Keywords:** essential hypertension, child, left ventricular hypertrophy, blood pressure load, dipping pattern

## Abstract

**Objective:**

To explore the association of dipping pattern and blood pressure load with left ventricular hypertrophy (LVH) in pediatric essential hypertension.

**Materials and Methods:**

Through an echocardiography monitor and an ambulatory blood pressure monitor of 425 children and adolescents diagnosed with essential hypertension with no treatment received, we identified 140 cases of LVH. Grouping patients according to LVH (LVH, *N* = 140; n-LVH, *N* = 285), we further evaluated their ambulatory blood pressure monitoring (ABPM) parameters by comparing dipping patterns between groups. A multivariable logistic regression analysis was used to determine the effect of blood pressure load on LVH.

**Results:**

No significant difference was found in systolic or diastolic blood pressure dipping patterns between groups (*P* = 0.161, *P* = 0.139). However, compared to the n-LVH group, the LVH group presented significant elevated nighttime systolic blood pressure (SBP) (*P* < 0.05), while nighttime DBP remained stable (*P* = 0.391), resulting in higher daytime and nighttime SBP loads, higher nighttime DBP load, and higher 24-h SBP load (*P* < 0.05). Notably, our multivariable logistic regression has shown that this trend of 24-h SBP load acts independently as a critical risk factor for LVH.

**Conclusion:**

Collectively, we observed a correlation between BP load and LVH in pediatric hypertension. Our data demonstrated that SBP load has a more significant weight in LVH progression, and 24-h SBP load, in particular, acts as a critical early prognostic parameter for LVH in pediatric hypertension.

## Introduction

Left ventricular hypertrophy (LVH) is the most common subclinical heart damage in pediatric essential hypertension (1, 2). Recent studies have indicated that LVH in pediatric hypertension can potentially cause the progression of cardiovascular risk when patients transit into adulthood (1, 3). Therefore, identifying early prognostic parameters associated with LVH in those hypertensive children and adolescents can help reduce their cardiovascular risk in adulthood. Blood pressure varying within 24 h was concluded as a circadian pattern (4). Fluctuation of blood pressure is one of the common causes of LVH as a long-term abnormal variation of blood pressure can induce hemodynamic overload of the cardiovascular system (5). Ambulatory blood pressure monitoring (ABPM) can record blood pressure and pulse in real-time and provide analysis of those recordings (6). In clinical practices, ABPM is used as a non-invasive approach to assess the overall level of blood pressure in pediatric essential hypertension (7). Studies have shown that ABPM results are more reliable and accurate than single clinic blood pressure (5, 7). The recent mechanistic hypothesis poses the basis for the association between ABPM parameters and LVH in hypertensive adults (8). Normally, blood pressure decreases about 10∼20% during sleeping, which is called the dipping pattern (4, 9). In hypertensive adults, reduction of nocturnal blood pressure increases the morbidity and mortality of cardiovascular diseases (10). Patients with the absence of a dipping pattern can have higher levels of the left ventricular mass index (LVMI) (11). Moreover, ABPM can be used to evaluate the blood pressure load and the percentage of abnormally elevated BP readings, which acts as an indicator of the cardiovascular system load. Some studies found that systolic blood pressure load is associated with LVH in hypertensive adults (12, 13). However, there are very limited data concerning the association between the dipping pattern and blood pressure load with LVH in pediatric essential hypertension. We carried out this case-control study to ascertain whether dipping pattern and blood pressure load are associated with LVH in pediatric essential hypertension and, more importantly, to identify which specific parameter(s) can potentially be used as early prognostic parameters for LVH in pediatric hypertension cases.

## Materials and Methods

### Study Population

Through a retrospective case-control study of the clinical database, 441 hypertensive children who were first diagnosed with essential hypertension from Children’s Hospital afflicted to Capital Institute of Pediatrics between August 2018 and August 2021, were selected. The database was then interrogated to extract data from 425 patients according to the following criteria. The inclusion criteria were as follows: (A) the diagnosis and the stage of hypertension evaluated according to “2018 Chinese Guidelines for Prevention and Treatment of Hypertension” (14); (B) children under the age range of 6–18 years old; (C) no former treatment received; and (D) a completed echocardiography data during the first hospitalization to assess left ventricular configuration changes. All the patients were inquired about their past medical history and were asked to complete the following tests, including, physical examination, blood testing (urea, creatinine, albumin, thyroid-stimulating hormone, aldosterone, or cortisol, and urine vanillylmandelic acid), 24-h urine protein testing, ultrasound of kidney, aorta, carotid artery, and electroencephalogram. We excluded those patients that were diagnosed with secondary hypertension caused by renal diseases, renal vascular diseases, endocrine diseases, vascular diseases, neurological disorders, or drugs. General data of patients were collected. Among the independent variables considered in this cohort, obesity and overweight are defined according to body mass index (BMI) cutoffs for overweight and obesity in Chinese children and adolescents aged 2–18 (15), Parental hypertension is defined as at least one parent diagnosed with hypertension. Informed consent was obtained from each patient and all parents and legal guardians, and the study was approved by the ethics committee of the Capital Institute of Pediatrics, Beijing, China (No: SHERLL2019003).

### Left Ventricular Hypertrophy and Classification

We chose the same method that our team used before to evaluate the left ventricular configuration and used the Devereux formula to calculate left ventricular mass (LVM) (16, 17). Left ventricular hypertrophy (LVH) was diagnosed according to the level of the left ventricular mass index (LVMI) and relative wall thickness (RWT), which were matching alternatives: (a) LVMI of ≥37.08 g/m^2.7^ in boys or LVMI of ≥34.02 g/m^2.7^ in girls; (b) RWT > 0.36. All individuals were divided into two groups: the LVH group (*n* = 140) and the n-LVH group (*n* = 285). Patients in the LVH group were divided into three groups according to their cardiac geometry: the concentric remodeling (CR) group with a normal level of LVMI and increased RWT, *n* = 64; the eccentric hypertrophy (EH) group with increased LVMI and a normal RWT, *n* = 39; and the concentric hypertrophy (CH) group with increased LVMI and RWT (17, 18).

### Ambulatory Blood Pressure Measurement

All children underwent 24-h ABPM (DMS-ABP, DM Software Inc., Beijing, China) (19). The device was set to record BP and heart rate (HR) every 30 min, and the monitoring should last at least 23 h. A measurement was considered reliable when the percentage of daytime/nighttime valid recording was more than 70% (20). Otherwise, ABPM should be performed again. Patients need to be quiet and inactive when the device is inflated. To avoid interference, patients need to stop any drug intake for at least 5 half-lives, which may influence blood pressure. Additionally, strenuous exercise, alcohol, or caffeine intake, as well as other activities that, may affect autonomic nervous function, should also be stopped (20). Through this retrospective study, we could not obtain the diary from the day the patient underwent ABPM. Since the latest guideline for pediatric hypertension [2017 AAP guideline (21)] did not mention the definition of daytime and nighttime, we chose the clock-based definition of daytime and nighttime recommended by the Hypertension, brain, cardiovascular and renal Outcome Prevention and Evidence in Asia (HOPE Asia) guidelines (9). The guideline could be more consistent with our patients in terms of ethnicity and living habits. On account of 2019 HOPE recommendations (9, 22), daytime was defined as the time between 09:00 and 21:00 h, and nighttime was defined as the time from 01:00 to 06:00 h. Therefore, 24-h, daytime, and nighttime BPs were averaged over the corresponding time.

### Definition

A dipping pattern is defined as a nocturnal BP fall between 10 and 20%. The loss of dipping pattern is commonly defined as a nocturnal BP fall (23), which is calculated as (daytime BP - nighttime BP)/daytime BP × 100 (21). Blood pressure load is the percentage of records in all if BP values reach or exceed sex-, age-, and height- specific 95% CI values (3).

### Statistics Analysis

All analyses were performed using SPSS 22.0. Continuous variables were checked for normal distribution by the Shapiro–Wilk test. Normally distributed variables were reported as mean ± SD and compared using an independent sample *t*-test or analysis of variance (ANOVA). Abnormally distributed variables were reported as median (Q1, Q4) and were compared using the Mann–Whitney *U* test or Kruskal–Wallis test. Categorical variables were reported as percentages and compared using the chi-square (χ2) analyses. Since BP load was abnormally distributed, correlation analysis between BP load, LVMI, and RWT was performed using Pearson’s correlation coefficient. Multivariable logistic regression models were used to evaluate the risk factors, in which we classified BP load by 25 and 50% according to the definition of ambulatory hypertension and other studies (9, 13, 22). A *p*-value of <0.05 was considered significant.

## Results

### General Characteristics of the Population

After excluding 17 individuals because of low-quality ABPM and age, there were a total of 425 participants in this study, including 331 men (77.9%) and 94 women (22.1%) with a mean age of 12.95 ± 2.26 years old. Based on their BMI, 304 participants (71.5%) were classified as obese and 74 children (17.45%) were overweight. A total of 210 participants (49.4%) were diagnosed with hypertension in stage 2 and 215 (50.6%) in stage 1. The mean clinical SBP and DBP of all participants were 137.72 ± 13 and 80.49 ± 11.43 mmHg, respectively. In addition, parents of 161 children have hypertension.

### Evaluation of Left Ventricular Hypertrophy

The mean LVMI of 425 patients was 30.29 ± 6.83 g/m^2.7^ and the mean RWT was 0.33 ± 0.05. A total of 140 patients (32.9%) were diagnosed with LVH, including 64 with CR (15.1%), 39 with EH (9.2%), and 37 with CH (8.7%). The characteristics of the two groups are presented in Table 1. Higher percentages of obesity and stage 2 hypertension were observed in the LVH group (*P* < 0.05). Characteristics among three abnormal cardiac geometry groups are shown in Table 2, where no statistical differences were observed.

**TABLE 1:** Characteristics of left ventricular hypertrophy (LVH) group and n-LVH group.

Patient characteristics	LVH, *N* = 140	n-LVH, *N* = 285	χ^2^	*P*
Boys (*n*, %)	117, 83.6 (%)	214, 75.1 (%)	3.923	0.062
Age (y)	13.02 ± 2.33	12.96 ± 2.28	–40.41	0.682
Parents hypertension (*n*, %)*^a^*	62, 55.4 (%)	99, 44.6 (%)	3.454	0.063
**Body size (*n*, %)**				
Obesity	112, 80 (%)	192, 67.4 (%)	11.1	0.004
Overweight	22, 15.7 (%)	52, 18.2 (%)		
Normal	6, 4.3 (%)	41, 14.4 (%)		
Hypertension stage 2 (*n*, %)	81, 57.9 (%)	129, 45.3 (%)	5.957	0.015

*Except for those given as percentages, values are mean ± SD.*

*LVH, left ventricular hypertrophy; n-LVH, not left ventricular hypertrophy.*

*^a^Family hypertension history was available in 334 patients.*

**TABLE 2:** Characteristics of groups among LVH.

	CR, *N* = 64	EH, *N* = 39	CH, *N* = 37	χ *^2^*	*P*
Boys (*n*, %)	55, 85.9 (%)	32, 82.1 (%)	30, 81.1 (%)	0.494	0.839
Age (y)	12.42 ± 2.2	13.00 ± 2.4	13.49 ± 2.36	2.605	0.078
Parents hypertension (*n*, %)*^a^*	31, 62 (%)	15, 46.9 (%)	16, 53.3 (%)	1.874	0.423
**Body size (*n*, %)**					
Obesity	47, 73.4 (%)	33, 84.6 (%)	32, 86.5 (%)	5.225	0.271
Overweight	13, 20.3 (%)	6, 15.4 (%)	3, 8.1 (%)		
Normal	4, 6.3 (%)	0, 0 (%)	2, 5.4 (%)		
Hypertension stage 2 (*n*, %)	38, 59.4 (%)	21, 53.8 (%)	22, 59.5 (%)	0.357	0.861

*Except for those given as percentages, values are mean ± SD.*

*CR, concentric remodeling; EH, eccentric hypertrophy; CH, concentric hypertrophy.*

*^a^Family hypertension history was available in 62 patients.*

### Dipping Pattern and Left Ventricular Hypertrophy

The percentage of SBP dipping pattern was 49.3% in the LVH group and 42.1% in the n-LVH group. The percentage of DBP dipping pattern was 42.9% in the LVH and 44.6% in the n-LVH group. There were no statistical differences between groups for SBP or DBP dipping pattern (*P* = 0.161, *P* = 0.139). After comparing the prevalence of dipping patterns among CR, EH, and CH groups, no statistical differences were found among groups either (53.1% vs. 41% vs. 51.4% in SBP, *P* = 0.481; 51.6% vs. 28.2% vs. 43.2% in DBP, *P* = 0.07). We also compared the nighttime BP level and observed that nighttime SBP was elevated in the LVH group (119.78 ± 10.98 vs. 117.18 ± 11.08 mmHg, *P* < 0.05), while nighttime DBP was not (65.4 ± 8.63 vs. 64.64 ± 8.50 mmHg, *P* = 0.391).

### Blood Pressure Load and Left Ventricular Hypertrophy

We calculated BP load at different points for LVH and n-LVH groups (Table 3). Significantly higher 24-h SBP load, daytime SBP load, nighttime SBP load, and nighttime DBP load were found in the LVH group (*P* < 0.05). In patients who are diagnosed with LVH (Table 4), we found no significant association between any BP load measurement and abnormal cardiac geometry (*P* > 0.05).

**TABLE 3:** The association of blood pressure load with left ventricular hypertrophy.

Blood pressure load (%)	LVH, *N* = 140	n-LVH, *N* = 285	*H*	*P*
24-h SBP load	50 (31, 71)	41 (20, 62.5)	2.614	0.009
Daytime SBP load	61.5 (37.25, 83)	52 (23, 74.5)	2.471	0.013
Nighttime SBP load	20 (0, 40)	0 (0, 33)	1.978	0.048
24-h DBP load	24.5 (13.25, 39)	24 (11, 42.5)	0.338	0.735
Daytime DBP load	29.5 (16.25, 50)	30 (14, 52)	0.055	0.956
Nighttime DBP load	0 (0, 20)	0 (0, 17)	5.957	0.04

*Values are given as median (Q1, Q4). SBP, systolic blood pressure; DBP, diastolic blood pressure; LVH, left ventricular hypertrophy; n-LVH, not left ventricular hypertrophy.*

**TABLE 4:** The association of blood pressure load with cardiac geometry.

Blood pressure load (%)	CR, *N* = 64	EH, *N* = 39	CH, *N* = 37	*H*	*P*
24-h SBP load	50 (31, 71.75)	53 (23, 71)	50 (35, 67)	0.051	0.975
Daytime SBP load	59.5 (38.75, 82.75)	65 (33, 84)	64 (39, 83)	0.234	0.89
Nighttime SBP load	20 (0, 40)	20 (0, 50)	20 (0, 45)	0.492	0.782
24-h DBP load	28.5 (13.25, 45)	21 (9, 32)	24 (12.5, 34)	2.628	0.269
Daytime DBP load	34.5 (17, 51.50)	25 (14, 40)	29 (17, 49)	2.790	0.248
Nighttime DBP load	0 (0, 20)	0 (0, 20)	0 (0, 10)	2.65	0.266

*Values are given as median (Q1, Q4). SBP, systolic blood pressure; DBP, diastolic blood pressure; CR, concentric remodeling; EH, eccentric hypertrophy; CH, concentric hypertrophy.*

As shown in Supplementary Figures 1A–E, 24-h SBP load and daytime SBP load were positively correlated with LVMI (*r* = 0.163, *P* = 0.001; *r* = 0.163, *P* = 0.001) and RWT (*r* = 0.149, *P* = 0.002; *r* = 0.149, *P* = 0.002). Nighttime DBP load was positively associated with RWT (*r* = 0.128, *P* < 0.05).

### Multivariable Logistic Analysis of Left Ventricular Hypertrophy

The single-factor analysis showed that obesity, hypertension stage, 24-h SBP load, and daytime SBP load were markedly different between the LVH and the n-LVH group (Table 5). Therefore, we included sex, age, obesity, hypertension stage, 24-h SBP load, and daytime SBP load in the stepwise regression analysis. As shown in Table 6, sex, obesity, and 24-h SBP load were considered independent risk factors for LVH.

**TABLE 5:** The univariate analysis of left ventricular hypertrophy.

Variables		*N*	LVH (%)	χ*^2^*	*P*
Sex	Male	94	24.5	3.923	0.062
	Female	331	35.3		
Age	<12 years	99	34.3	0.115	0.807
	≥12 years	326	32.5		
Obesity	Yes	304	36.8	11.1	0.004
	No	121	23.1		
Hypertension stage	Stage 1	215	27.4	5.957	0.015
	Stage 2	210	38.6		
SBP dipping pattern	Yes	189	36.5	1.96	0.178
	No	236	30.1		
DBP dipping pattern	Yes	187	32.1	0.111	0.756
	No	238	33.6		
24-h SBP load	0∼25%	112	23.2	6.706	0.035
	25∼50%	134	35.1		
	>50%	179	37.4		
Daytime SBP load	0∼25%	98	20.4	9.081	0.011
	25∼50%	97	36.1		
	>50%	230	37		
Nighttime SBP load	0∼25%	289	31.1	3.296	0.195
	25∼50%	73	31.5		
	>50%	63	42.9		
24-h DBP load	0∼25%	225	31.6	0.62	0.743
	25∼50%	138	35.5		
	>50%	62	32.3		
Daytime DBP load	0∼25%	189	32.3	2.12	0.355
	25∼50%	128	37.5		
	>50%	108	28.7		
Nighttime DBP load	0∼25%	364	31.3	5.086	0.084
	25∼50%	41	36.6		
	>50%	20	55		

*LVH, left ventricular hypertrophy; SBP, systolic blood pressure; DBP, diastolic blood pressure.*

**TABLE 6:** Independent risk factors for left ventricular hypertrophy.

Risk factors	OR	95% CI		*P*
Male sex	1.792	1.052	3.052	0.032
Obesity	1.653	1.157	2.360	0.006
24 h SBP load	1.381	1.057	1.806	0.018

*OR, odds ratio; CI, confidence interval; SBP, systolic blood pressure.*

## Discussion

Left ventricular hypertrophy is the major subclinical cardiovascular damage in children and adolescents with essential hypertension, and this cardiac geometry remodeling is an adaptive change of sustained high blood pressure load (5, 17). Previous studies found that the prevalence of left ventricular hypertrophy in pediatric hypertension is 20∼40% (1, 2, 5). In this study of 425 children diagnosed with essential hypertension, 32.9% of patients already had left ventricular hypertrophy diagnosed during their first visit, which indicated that subclinical cardiac damage has occurred before the first visit. Concentric remodeling, defined as a normal level of LVMI and increased RWT, was a common cardiac geometrical change (18). ABPM can be applied in evaluating various BP features including BP dipping pattern and BP load. Studies have found these two parameters associated with LVH in adults (10, 13), but little was documented on such matters in children and adolescents. Therefore, we aimed to clinically explore the association of these two ABPM determinants with left ventricular hypertrophy in a large sample of pediatric essential hypertension and expected that, by doing so, we could progress into performing better clinical practice in early diagnosis of LVH.

Circadian blood pressure has two peaks during daytime and falls during nighttime sleep (4). Since the latest guideline for pediatric hypertension [2017 AAP guideline (24)] did not specify the definition of daytime and nighttime, we applied the clock-based definition of daytime and nighttime recommended by the HOPE guideline (9). The guideline could be more suitable for our patients’ ethnicity and living habits. Endogenous and exogenous rhythms regulate 24-h BP fluctuation (4). The decline of BP during sleep is considered to be ambiguously associated with left ventricular hypertrophy in adults (17, 20, 22). Most researchers consider that the loss of dipping pattern may cause hypertensive target organ damage and increase other cardiovascular risks (11, 25, 26). A meta-analysis showed that nocturnal SBP fall provided prognostic information in hypertensive adults, in which there is a higher chance for cardiovascular events incidents to occur when the loss of dipping pattern was observed (10). To our knowledge, there are different opinions about the correlation between dipping patterns and LVH in hypertensive children and adolescents. In this study, we divided patients into two study groups according to the evaluation of whether a dipping pattern was observed, where a normal dipping pattern was established according to various relevant studies (10, 26, 27). However, after careful observation, we did not find any relevance between the dipping pattern and LVH. The dipping status is defined based on the rate of nocturnal fall, which corresponds to the ratio of nighttime and daytime BPs. Some studies suggest that nocturnal BP may be a better predictor than the dipping pattern of LVH (27, 28). We also compared nighttime BP in the two groups and observed that nighttime SBP was elevated in the LVH group, while nighttime DBP was not. The prevalence of dipping patterns was similar in the two groups, which indicated that elevated nighttime BP is not consistently accompanied by a change in the dipping status. The rate of nocturnal fall can remain 10–20% when both daytime BP and nighttime BP are elevated, which, by definition, can still be classified as a dipping pattern. Another research group has confirmed in their mild pediatric essential hypertension study that no lower diurnal BP fall was observed (29). A study conducted in the United Kingdom general adolescent population has drawn a similar conclusion (30). In summary, there has been no evidence of an observed correlation between nighttime dipping and cardiac structure changes. Therefore, we hypothesized that BP level and BP load could be more significant than the dipping pattern in the matter of LVH in pediatric essential hypertension.

Changes in left ventricular mass are directly impacted by hemodynamic load (18). Blood pressure load reflects the proportion of time when blood pressure increases above the normal level, which also indicates the chronic load of the cardiovascular system. Chronic overload of the cardiovascular system causes the formation of cardiac hypertrophy in patients with hypertension (31). It has been proven that SBP load is associated with LVH in adults (12, 13). However, there is no consistent conclusion regarding the relationship between BP load and LVH in children and adolescents. Jonathan et al. reported that LVMI correlated positively with 24-h SBP load and wake SBP load in 37 children with hypertension without secondary hypertension and 33 normotensive children (8). Moreover, they found a higher prevalence of LVH in patients with high systolic BP load. However, other researchers suggested that SBP load lacks value in prognosing adverse cardiac outcomes in children with hypertension with chronic kidney disease (24). Our study observed that 24-h SBP load, daytime SBP load, nighttime SBP load, and nighttime DBP load are higher in the LVH group. Also, 24-h SBP load and daytime SBP load are positively correlated with LVMI and RWT. The results showed an association of blood pressure load, especially systolic blood pressure load, with LVH. Current guidelines have demonstrated the importance of ABPM in pediatric hypertension for its role in diagnosis, evaluation, and therapeutic management (14, 21). Based on the results obtained in this study, we are confident about the necessity of ABPM and the importance of subsequent BP load in pediatric essential hypertension.

Multivariate analysis found that male sex, obesity, and 24-h SBP load were independent risk factors for LVH in pediatric essential hypertension. Previous studies on Chinese children and adolescents revealed that boys have shown a higher prevalence of high BP (32). In our study, 77.9% of the participants were boys, so we raised a hypothesis that male sex contributes as an independent risk factor of LVH. In addition, obesity is common in pediatric essential hypertension. Previous studies confirmed that obesity and ambulatory SBP are independently associated with cardiac hypertrophy in children (32, 33). Our research reached a similar conclusion. Thus, BP control and weight loss should both be considered in clinical blood pressure management. In addition, we found that ambulatory 24-h SBP load is independently associated with LVH after considering the effect of sex, age, obesity, hypertension stage, and DBP load, which suggested that systolic BP elevation has a significant impact on LVH. Moreover, this observation demonstrates that systolic BP elevation is a characteristic of essential hypertension in children. Future studies should focus on investigating long-acting antihypertensive treatment to control systolic BP in pediatric hypertension cases regardless of patients’ left ventricular hypertrophy status. Left ventricular geometrical change is classified by LVMI and RWT into three abnormal types. Since we found that daytime systolic is positively correlative with LVMI and RWT and nighttime diastolic load is positively correlative with RWT. Personalized anti-hypertensive treatment should take time of BP load into consideration.

## Conclusion

The blood pressure dipping pattern was observed to have no influence on cardiac hypertrophy in pediatric essential hypertension. However, blood pressure load was observed to be associated with left ventricular hypertrophy. Notably, a 24-h SBP load acts independently as a risk factor for left ventricular hypertrophy in pediatric essential hypertension. Therefore, our study proved the importance of being vigilant about blood pressure load in patients with pediatric essential hypertension in clinical practice–closely monitoring patients’ ambulatory blood pressure can contribute to early discovery of cardiac hypertrophy and immediate sensible antihypertensive treatment plans for patients.

## Limitations

Since the ABPM was performed during hospitalization and patients were not in their natural environment during the assessment, the white-coat effect should also be taken into account. Our hypertension ward provides a relaxed environment as far as possible to reduce this effect in this study.

## Data Availability Statement

The raw data supporting the conclusions of this article will be made available by the authors, without undue reservation.

## Ethics Statement

The studies involving human participants were reviewed and approved by the Ethics Committee of Children’s Hospital Affiliated to Capital Institute of Pediatrics, Beijing, China. Written informed consent to participate in this study was provided by the participants’ legal guardian/next of kin.

## Author Contributions

TZ: data collection. HW: writing–original draft. HW, YL, and LS: writing–review and editing. LS: supervision. All authors contributed to the article and approved the submitted version.

## Conflict of Interest

The authors declare that the research was conducted in the absence of any commercial or financial relationships that could be construed as a potential conflict of interest.

## Publisher’s Note

All claims expressed in this article are solely those of the authors and do not necessarily represent those of their affiliated organizations, or those of the publisher, the editors and the reviewers. Any product that may be evaluated in this article, or claim that may be made by its manufacturer, is not guaranteed or endorsed by the publisher.
