# Supplementary material for: The Correlation Between ABPM Parameters and Left Ventricular Hypertrophy in Pediatric Essential Hypertension
Source: Front Pediatr. 2022 Jun 2;10:896054. doi: 10.3389/fped.2022.896054 (PMC9201109; doi:10.3389/fped.2022.896054)
Supplement: Supplementary file 1 [file Data_Sheet_1.docx]

Supplementary Material

# Supplementary Figures

## Supplementary Figures

| 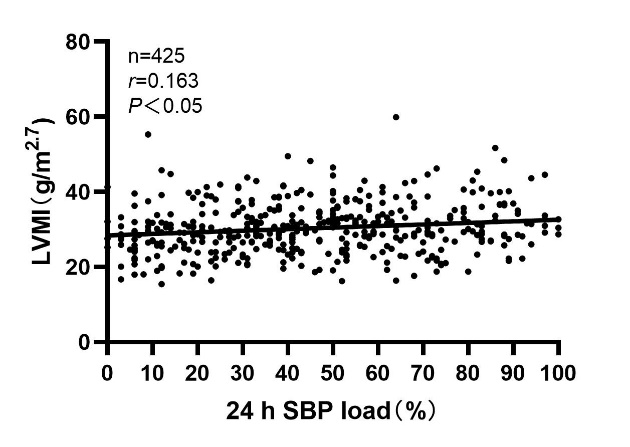  **A** | 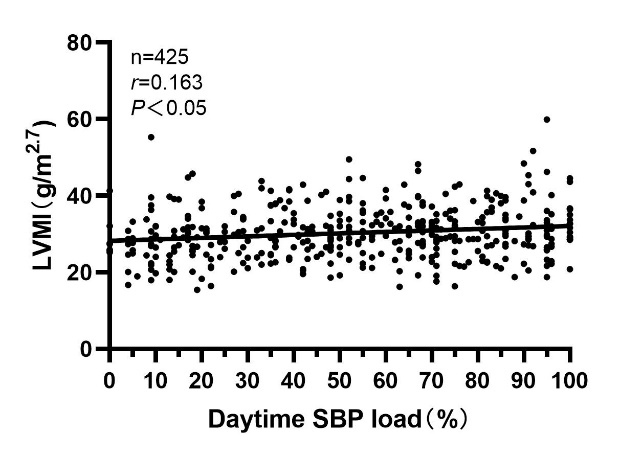  **B** |
| --- | --- |
| 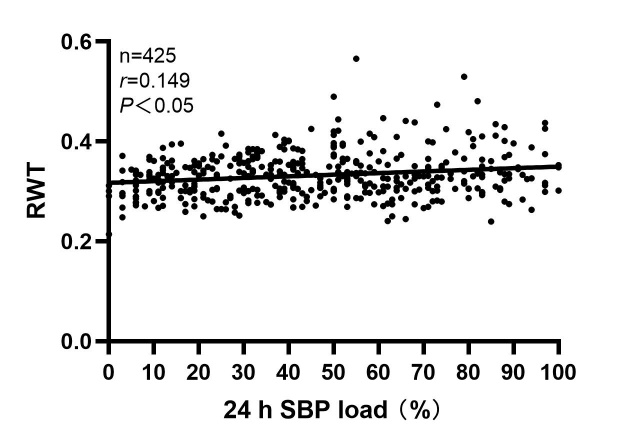  **C** | 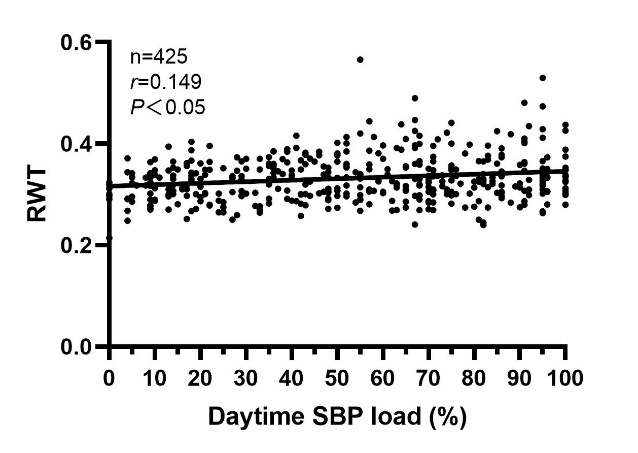  **D** |
| 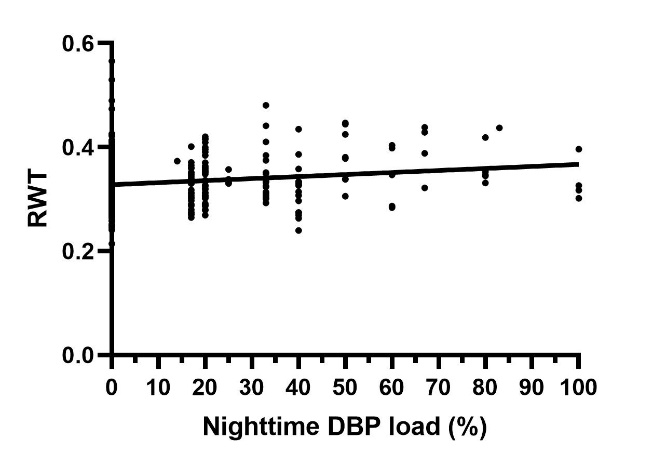  **E** |  |

**Figure 1** Scatter plot of correlation between left ventricular hypertrophy and blood pressure load

(A) Scatter plot of correlation between LVMI and 24 h SBP load;

(B)Scatter plot of correlation between LVMI and daytime SBP load;

(C) Scatter plot of correlation between RWT and 24 h SBP load;

(D) Scatter plot of correlation between RWT and daytime SBP load;

(E) Scatter plot of correlation between RWT and nighttime DBP load.

(LVMI, left ventricular mass index; RWT, relative wall thickness; SBP, systolic blood pressure; DBP, diastolic blood pressure)
